# The Interplay between Maternal Depression and ADHD Symptoms in Predicting Emotional and Attentional Functioning in Toddlerhood

**DOI:** 10.1007/s10802-025-01332-y

**Published:** 2025-05-19

**Authors:** Michal Levy, Andrea Berger, Alisa Egotubov, Avigail Gordon-Hacker, Eyal Sheiner, Noa Gueron-Sela

**Affiliations:** 1 Department of Psychology, Ben-Gurion University of the Negev, Be’er Sheva, Israel; 2 School of Brain Sciences and Cognition, Ben-Gurion University of the Negev, Beer Sheva, Israel; 3 Department of Obstetrics and Gynecology, Soroka University Medical Center, Beer-Sheva, Israel

## Abstract

Depression and ADHD frequently co-occur, presenting unique challenges such as difficulties in executive functioning and reduced quality of life. Both conditions are also associated with less optimal parenting behaviors. Despite the prevalence of this comorbidity, the combined effects of maternal depressive symptoms and ADHD on child development remain underexplored, particularly in early childhood, a critical period marked by heightened brain plasticity and reliance on external regulation. This study examines how maternal depression and ADHD symptoms jointly predict children’s emotional and attentional development across the first two years of life. A sample of 156 mothers and children (51% boys) participated in a three-wave longitudinal study from pregnancy to two years postpartum. Maternal ADHD symptoms were reported during pregnancy, while depressive symptoms were reported at pregnancy, three months, and two years postpartum. At two years of age, child depressive symptoms were reported by mothers, and focused attention was observed during a structured play session. Findings revealed that the combination of high maternal ADHD and depressive symptoms in the perinatal period predicted the most significant difficulties in children at two years of age, including elevated depressive symptoms and reduced focused attention. Specifically, maternal depressive symptoms were associated with greater child depressive symptoms and lower focused attention only when maternal ADHD symptoms were elevated. These results highlight the compounding effects of maternal psychopathology on child development, emphasizing the importance of targeted support for children at heightened risk of emotional and cognitive impairments due to parental psychopathology.

**Supplementary Information:**

The online version contains supplementary material available at 10.1007/s10802-025-01332-y.

In recent years, research has increasingly highlighted the impacts of parental mental health on child development, particularly in relation to parental depression (Goodman et al., 2011) and attention-deficit/hyperactivity disorder (Friedrich et al., 2017; Johnston & Chronis-Tuscano, 2017). Depression and ADHD frequently co-occur (for a review, see Choi et al., 2022), and individuals with both conditions tend to face greater challenges, including lower quality of life and higher levels of physical illness (McIntyre et al., 2010). Despite these findings, the unique and combined effects of parental depressive symptoms and ADHD on child outcomes remain underexplored, particularly among young children (Brammer et al., 2018; Smit et al., 2021). Understanding the interplay between maternal depression and ADHD during early childhood is crucial, as this developmental period is characterized by heightened brain plasticity and a significant reliance on external regulation of affect and behavior (Kolb & Gibb, 2011; Sameroff, 2010; Tierney & Nelson, 2009). Moreover, recognizing and identifying infant mental health difficulties early on can facilitate timely interventions, potentially preventing long-term adverse effects (Clinton et al., 2016; Izett et al., 2020).

This study seeks to address this gap by examining the incremental and interactive contributions of maternal perinatal depression and ADHD symptoms to child outcomes, specifically depressive symptoms and focused attention abilities, in toddlerhood. By investigating these relationships from pregnancy through the first two years of life, this research aims to shed light on the early mechanisms underlying the intergenerational transmission of depression and ADHD.

## Maternal Depression and ADHD in the Context of Parenting

Depression or major depressive disorder (MDD) is a mood disorder characterized by depressed feelings, loss of interest or pleasure, pessimistic thoughts about the self and the future, and physiological symptoms such as disturbed sleep or appetite (American Psychiatric Association, 2013). The prevalence of MDD among mothers during early and middle childhood ranges between 10 and 19% (Ertel et al., 2011; Gelaye et al., 2016). Mothers experiencing depression often display maladaptive parenting behaviors, including disengagement from their child and negative interactions, such as hostility and negative maternal affect (Lovejoy et al., 2000). Additionally, elevated maternal depressive symptoms (MDS) have been associated with increased risk for a range of child cognitive and socioemotional adjustment problems, from emotional and behavioral difficulties, such as internalizing and externalizing problems, as well as negative affect and behavior (for a meta-analysis, see Goodman et al., 2011), to cognitive impairments that together with other contextual factors are related to lower language skills and IQ (for a review, see Grace et al., 2003).

Goodman and Gotlib’s (1999) model of risk transmission in children of depressed mothers highlights how maternal depression can be transmitted to children through multiple etiological mechanisms, including those operating during the prenatal and postnatal periods. Specifically, prenatal maternal depression may disrupt fetal neurodevelopment through abnormal neuroendocrine functioning, exposing the fetus to dysregulated hormonal processes, which may contribute to later emotional and behavioral difficulties (Goodman & Gotlib, 1999). For instance, prenatal depression can lead to elevated maternal cortisol levels, which may then contribute to heightened emotional reactivity in offspring (Swales et al., 2018). In contrast, postnatal depression can primarily affect the child through disruptions in the caregiving environment. Depressed mothers often struggle to engage effectively with their children, impacting their role as social partners. For example, they may struggle with attention-directing strategies (Goldsmith & Rogoff, 1997), be less sensitive in interactions (Bernard et al., 2018), or exhibit intrusive behaviors (Gueron-Sela et al., 2018), all of which can hinder children’s ability to sustain attention. As a result, the child may receive inadequate social, cognitive, and emotional support, ultimately leading to an increased risk of developing depressive symptoms and impaired cognitive and attentional skills (Goodman & Gotlib, 1999). Indeed, both prenatal and postnatal maternal depression have been linked to increased behavioral problems in children (Barker et al., 2011), and has also been identified as a risk factor for offspring depression (Pearson et al., 2013).

This is especially relevant when considering maternal postpartum depression, which differs from longer histories of depression. Postpartum depression has been found to specifically affect key caregiving activities, such as feeding practices (especially breastfeeding), sleep routines, well-child visits, vaccinations, and safety measures, making this period particularly sensitive and impactful (Field, 2010). Additionally, a child’s age plays a critical role in their vulnerability to maternal depression (Goodman et al., 2011). During early childhood, children undergo a particularly sensitive developmental period, requiring greater external regulation and maternal support, which makes them more vulnerable to psychopathology (Goodman et al., 2011; Sameroff, 2010). Specifically, in early infancy, children are highly dependent on their caregivers for the regulation of affect, sleep, and feeding. Parenting during this stage is primarily characterized by sensitive and contingent responses to the infant’s cues, which lay the foundation for the development of later emotional capacities (Feldman, 2007; Sameroff, 2010). As children enter toddlerhood, they begin to assert greater autonomy and develop foundational self-regulatory capacities. In response, parental behavior must adapt, placing greater emphasis on behavioral guidance, limit setting, and support for emerging independence (Kopp, 1982). Notably, within the first few years of life, infants transition from a state of high dependence on caregivers for regulation and support to increasing autonomy, necessitating adaptations in parenting behaviors to meet their evolving developmental needs (Goodman et al., 2011; Sameroff, 2010).

An additional form of psychopathology that may have a distinct and significant impact on children’s outcomes is parental ADHD. ADHD is a neurodevelopmental disorder characterized by symptoms of inattention, hyperactivity, and impulsivity (American Psychiatric Association, 2013), with a prevalence of approximately 7% among adults (Song et al., 2021). In the context of parenting, maternal ADHD symptoms, in particular, have been linked to challenges in parenting (Friedrich et al., 2017; Johnston & Chronis-Tuscano, 2017). For example, mothers with ADHD were found to be less effective in monitoring child behavior, less consistent in discipline, and had lower-quality problem-solving skills compared to mothers without ADHD (Murray & Johnston, 2006). In addition, parental ADHD symptoms were associated with less positive and more harsh and lax parenting behaviors (Park et al., 2017). Mothers with ADHD also reported experiencing more postpartum depressive symptoms and greater hostility toward their infant five months after birth, underscoring the potential interactions between parental ADHD, depression, and attachment, which may subsequently influence child depressive symptoms (Joseph, 2023). These factors may impair a mother’s ability to engage in responsive and structured interactions with her child, leading to a parenting style that is more reactive, unpredictable, and emotionally disengaged. As a result, children may experience inconsistent caregiving, increased stress, and reduced opportunities for developing essential emotional and behavioral regulation skills, ultimately heightening their risk for emotional and behavioral difficulties (Christaki et al., 2022; Goodman & Gotlib, 1999; Park et al., 2017).

### The Interplay between Maternal Depression and ADHD

Studies show a high comorbidity between ADHD and depression during adulthood, with the prevalence of depressive disorders among individuals diagnosed with ADHD ranging from 9 to 55%, compared to only 1–12% in those without an ADHD diagnosis (for a review, see Choi et al., 2022). While ADHD and depression share certain characteristics, such as difficulties in concentration (American Psychiatric Association, 2013), studies also highlights key differences (Humphreys et al., 2012; Smit et al., 2021). Maternal depression has been associated with greater affective difficulties, lower quality of life, challenges in social relationships, and insecure attachment with offspring (Slomian et al., 2019). In contrast, mothers with ADHD tend to exhibit higher levels of disorganization, reduced involvement, and inconsistent parenting practices (Chronis-Tuscano et al., 2008; Friedrich et al., 2017). These patterns have been linked to an increased risk of externalizing behaviors and attentional difficulties in children (Efron et al., 2018).

While depression and ADHD have both been related to less optimal parenting behaviors (Goodman et al., 2011; Johnston & Chronis-Tuscano, 2017), only a few studies have investigated the interactions between parental depression and ADHD in the context of parenting and child outcomes. A recent cross-sectional study examining families with children aged 6–11 found that parental depressive symptoms were linked to more negative and fewer positive self-reported parenting behaviors, while parental ADHD symptoms do not show the same pattern (Smit et al., 2021). However, the study focused on the links between parental depression and ADHD and parenting behaviors, without directly examining predictions for child outcomes. Another study explored how changes in parental ADHD and depression influenced child ADHD, finding that reductions in parental ADHD, but not depression, were significantly associated with reduced ADHD symptoms in children (Brammer et al., 2018). However, this study focused solely on adolescent ADHD and did not examine interactive associations between parental ADHD and depression. The literature examining the combined effects of parental ADHD and depression on outcomes in young children is notably limited. Parents with both depression and ADHD may face compounded challenges including difficulties in executive functioning (Bain, 2018), reduced quality of life, increased interpersonal difficulties (Huang et al., 2022) as well as more severe depressive symptoms, higher rates of suicidality, and increased hospitalization (Biederman et al., 2008). Understanding how these co-occurring psychopathologies influence parenting and child development is therefore critical. Research suggests that ADHD and depression are interrelated within families and should be examined together (Segenreich et al., 2015). According to the developmental-transactional model (Johnston & Chronis-Tuscano, 2015, 2017), the interplay of familial risk factors, such as parental psychopathology, shapes child outcomes. Parents with ADHD may develop coping strategies to manage executive function difficulties, such as using tools and routines to create external structure in daily life (Canela et al., 2017). Similarly, parents experiencing depression might rely on social support systems to help reduce the depressive symptoms (Gariépy et al., 2016). Parents who experience high levels of either ADHD or depression may develop strategies to mitigate the challenges associated with their condition, for instance, using strategies or tools to structure daily routines to address executive function deficits in the context of ADHD, or relying on social support to reduce depressive symptoms. However, when both disorders are present simultaneously, their ability to adapt may be compromised. For example, mothers with ADHD may face difficulties in maintaining consistent routines (American Psychiatric Association, 2013), while co-occurring depression can limit emotional availability and responsiveness (Bernard et al., 2018; Gueron-Sela et al., 2018). The cumulative cognitive and emotional burden associated with managing both conditions may hinder their ability to effectively cope with either (Dean et al., 2022), thereby amplifying parenting challenges. Together, these difficulties may result in greater disruptions to parenting behaviors and child development that exceed the combined impact of each condition alone (Smit et al., 2021). Given these challenges and considering the high rates of comorbidity between depression and ADHD, it is critical to better understand how the interplay between these forms of psychopathology is associated with children’s development.

## Depressive Symptoms in Early Childhood

Depressive behaviors in children can emerge as early as 24 months and may include symptoms like tearfulness, irritability, lack of joy, sadness, and loss of interest in play (Luby & Belden, 2012). Depression in early childhood can have significant implications later in life, such as poorer mental health and lower educational achievement (Dekker et al., 2007), decreased social support (Psychogiou et al., 2024), and enduring depressive symptoms, making it essential to understand their etiology. These symptoms can create considerable challenges for children and their families, interfering with their daily lives and overall well-being (Ogburn et al., 2010). Furthermore, early identification of infant mental health difficulties enables timely interventions, potentially mitigating long-term adverse effects (Clinton et al., 2016; Izett et al., 2020).

One of the strongest predictors of early childhood depression is maternal depression. Maternal depressive symptoms, both during pregnancy and postpartum, have been shown to predict children’s depressive symptoms (Kingston et al., 2018), particularly when experienced in both periods (Lahti et al., 2017). The integrative model for the transmission of risk to children of depressed mothers (Goodman & Gotlib, 1999), provides a framework for understanding how MDS can influence and be transmitted to children. This model highlights mechanisms such as genetic factors, exposure to maladaptive or negative maternal behaviors, thoughts, and emotions, along with other maternal and child characteristics (Goodman & Gotlib, 1999). For instance, evidence from twin studies, genetic predispositions, gene variants, and hormonal factors like cortisol highlight the role of genetic heritability in this process (for a review, see Sawyer et al., 2019). Additionally, research has demonstrated the impact of exposure to maladaptive or negative parenting behaviors. For example, maternal depressive symptoms were linked to negative perceptions of the child and parenting practices (e.g., harsh, unresponsive, punitive), which, in turn, predicted depressive symptoms in children (Wang, 2018). To our knowledge, only one study examined the effects of both parental depression and ADHD symptoms on behavioral problems and ADHD symptoms in children aged 5–10 years. The findings indicated that parental depression was associated with greater child internalizing behaviors, while parental ADHD predicted child ADHD symptoms as well as internalizing and externalizing behaviors. However, this study did not investigate longitudinal or interactive effects between parental ADHD and depression (Humphreys et al., 2012).

## Focused Attention in Early Childhood

A key milestone in the development of attentional skills essential for goal-directed behavior is focused attention, which is the ability to sustain active engagement with a stimulus or a task over an extended period while disregarding irrelevant information and distractions (Garon et al., 2008; Ruff & Rothbart, 1996). Focused attention develops gradually during the early years of life, with notable progress occurring between 1 and 2 years and again between 2.5 and 3.5 years. This development is linked to the growing complexity and variety of activities children engage in, as well as improvements in their regulatory skills (Ruff & Lawson, 1990). Previous studies have identified focused attention as crucial, showing its positive association with self-regulatory functions (Johansson et al., 2015), executive functions (Garon et al., 2008), reading skills (Bigozzi et al., 2018), language development in toddlerhood and academic performance in adolescence (Burstein et al., 2024). Moreover, focused attention in the first year of life has been found to be negatively associated with hyperactivity and inattention problems reported in preschool (Lawson & Ruff, 2004), with low focused attention serving as a potential risk factor for ADHD. Research has shown that children’s focused attention abilities are shaped by multiple environmental factors (Gaertner et al., 2008). For example, maternal postpartum depression has been identified as a risk factor for ADHD in children (Christaki et al., 2022). Additionally, deficits in attention skills in children were found to be predicted by maternal internalizing symptoms (Klemp et al., 2023). One proposed mechanism is that mothers experiencing ADHD or depressive symptoms may have reduced capacity for positive and nurturing interactions with their children and may be at greater risk for more negative and inconsistent interactions, ultimately contributing to emotional and behavioral challenges in children (Christaki et al., 2022; Goodman & Gotlib, 1999; Park et al., 2017).

## The Current Study

The current study aimed to explore the interactive associations between maternal depressive and ADHD symptoms, commonly intertwined yet understudied risk factors, and children’s emotional and attentional outcomes. Given prior research linking maternal depression to a range of child outcomes (Goodman et al., 2011) and the emergent literature examining the combined effects of maternal depression and ADHD symptoms (Brammer et al., 2018; Smit et al., 2021), this study examined how these forms of psychopathology jointly predict children’s emotional and attentional development. Utilizing longitudinal data, we investigated these relationships from pregnancy through the first two years of life. Specifically, we hypothesized that:

1. Maternal depressive symptoms, both prenatal and postpartum, would predict children’s depressive symptoms and focused attention at 2 years of age. Specifically, higher maternal depressive symptoms would predict children’s higher depressive symptoms and lower focused attention.
2. Higher maternal ADHD symptoms would predict children’s lower focused attention and higher depressive symptoms at 2 years.
3. Mothers with co-occurring ADHD and depressive symptoms face amplified challenges, including impaired executive functioning (Bain, 2018), increased interpersonal difficulties (Huang et al., 2022) as well as more severe depressive symptoms (Biederman et al., 2008). Therefore, the interaction between elevated maternal ADHD and depressive symptoms is expected to serve as a dual risk factor, predicting the highest level of depressive symptoms and lowest level of focused attention in children. Specifically, when both maternal ADHD and depressive symptoms are high, children are expected to exhibit more depressive symptoms and reduced focused attention.

## Method

### Participants

The sample included families participating in a longitudinal study, following mothers and infants from the prenatal period through the first two postpartum years. Mothers were recruited during the second trimester of pregnancy through social media platforms and the obstetrics and gynecology division at Soroka Medical center. All women recruited were physically healthy, above 21 years of age, with no reported substance abuse. Post hoc exclusion criteria were preterm birth (< 37 weeks) or child/mother chronic health problems. For the current study, we used data collected at three time points: the second trimester of pregnancy (T1, *N* = 180), three months (T2, *N* = 139), and 24 months (T3, *N* = 146) of infants’ age. Date were collected between November 2018 to September 2022. The final sample included 156 mother-infant dyads who had data from at least two time points. Mothers excluded from the analyses were, on average, one year younger (*t =* -94.676, *p* <.000), had two fewer years of education (*t =* -71.499, *p* <.000), and were one week earlier (*t =* -83.329, *p* <.000) in their pregnancy compared to those in the final sample. No other differences were observed. Demographic information is reported in Table 1.

**Table 1:** Participants’ demographic characteristics

		T1	T2	T3
Age	*M (SD)*			
Mothers (Years)		30.9 (4.20)	-	-
Child (Months)		-	3.0 (0.73)	26.1 (1.98)
Pregnancy Week		*M (SD)*		
		21.3 (2.74)	-	-
Mothers’ Education	*n (%)*			
High-school diploma		21 (13.5%)	-	-
BA Academic degree		74 (47.4%)	-	-
MA Academic degree		37 (23.7%)	-	-
Ph.D. Academic degree		6 (3.8%)	-	-
Professional training		10 (6.4%)	-	-
Other		6 (3.8%)	-	-
Missing		2 (1.3%)	-	-
Number of children in the family	*n (%)*			
One child		65 (41.7%)	-	39 (25.0%)
Two children		54 (34.6%)	-	61 (39.1%)
Three children		25 (16.0%)	-	29 (18.6%)
Four children or more		10 (6.4%)	-	12 (7.7%)
Missing		2 (1.3%)		15 (9.6%)
Child Sex (boys)				
		-	80 (51.3%)	-
Marital Status	*n (%)*			
Married		140 (89.7%)	-	128 (82.1%)
Single		3 (1.9%)	-	3 (1.9%)
In a relationship		7 (4.5%)	-	7 (4.5%)
Divorced		1 (0.6%)	-	2 (1.3%)
Separated		1 (0.6%)	-	1 (0.6%)
Missing		4 (2.6%)	-	15 (9.6%)
Family monthly income (based on the national monthly average)			*n (%)*	
Much less than average		9 (6%)	-	-
Less than average		43 (28%)	-	-
Average		57 (37%)	-	-
More than average		36 (23%)	-	-
Much more than average		3 (2%)	-	-
Missing		8 (5%)	-	-
Mothers diagnosed with ADHD		22 (14.2%)	-	-
Mothers receiving medication for ADHD		1 (0.6%)	-	-
Mothers diagnosed with major depressive disorder		8 (5.2%)	-	-
Mothers receiving medication for major depressive disorder		1 (0.6%)	-	-

### Procedure

The study protocol was approved by the Institutional Review Board at Soroka Medical center. Written consent was obtained from all participants prior to data collection. Data for T1 were collected during a home visit while the mothers were pregnant. During this visit, they completed questionnaires on demographic information and self-reported ADHD symptoms. Additionally, mothers completed questionnaires evaluating their self-reported depression symptoms at each time point (T1-T3) and assessed their child’s depressive symptoms at T3. During the T3 home visit, children’s focused attention was recorded during an independent block play session. Focused attention was later rated off-line by trained research assistants.

### Measures

#### Maternal Depressive Symptoms

The Edinburgh Postnatal Depression Scale (EPDS; Cox et al., 1987) was administered at T1-T3 to assess the severity of self-reported maternal depressive symptoms. The EPDS consists of 10 items that primarily assess emotional aspects of postpartum depression (PDD; e.g., ‘In the past 7 days, how often did you feel so unhappy that you had difficulty sleeping?‘) and has also been validated for use with pregnant women (Levis et al., 2020). Mothers rated each item on a four-point Likert scale, and the scores for each time point were summed, with higher scores indicating greater levels of depressive symptoms (Cronbach’s α ranged from 0.81 to 0.84 across all time points). At T1, 29 mothers (19%) scored above the clinical cut-off (10 or above), while 14 mothers (9%) did so at T2, and 16 mothers (10%) at T3.

### Maternal ADHD Symptoms

To assess maternal ADHD symptoms at T1, mothers completed The World Health Organization Adult ADHD Self-Report Scale Screener (ASRS; Kessler et al., 2005), which consists of 6 items (e.g., ‘How often do you have difficulty getting things in order when you have to do a task that requires organization?’). Mothers rated each item on a five-point Likert scale, reflecting how they had felt and behaved over the past six months. The item scores were summed (Cronbach’s α = 0.83), with higher scores indicating greater ADHD symptoms in adulthood. Previous research (Bastiaens & Galus, 2018) identified a cutoff score of 12 or above as the most effective threshold. Using this cutoff, 28 mothers (18%) scored above the clinical threshold. Since mothers reported their ADHD symptoms during pregnancy, we also examined whether having a formal ADHD diagnosis was associated with their symptom reports on the ASRS. Mothers who reported an ADHD diagnosis had significantly higher scores on the ASRS screener (*t*_(152.97)_ = -23.80, *p* <.000) and were more likely to meet the clinical cutoff (*X*^*2*^ (1, *N* = 152) = 19.61, *p* <.000).

### Child Depressive Symptoms

Children’s depressive symptoms at T3 were assessed using the Child Behavior Checklist for Ages 1.5–5 (CBCL 1½–5; Achenbach & Rescorla, 2000). The Anxious/Depressed subscale, consisting of 8 parent-reported items, was used as an indicator of child depressive symptoms. Mothers rated how accurately each statement described their child’s behavior over the past two months on a three-point Likert scale ranging from 0 (Not true) to 2 (Very often true; e.g., ‘Nervous, high-strung, or tense’). Item scores were summed (Cronbach’s α = 0.71), with higher scores reflecting greater levels of depressive symptoms.

### Child Focused Attention

Children’s focused attention was assessed during an independent block play session using the LAB-TAB Locomotor Version (H. H. Goldsmith & Rothbart, 1999), drawing on prior research that employed this paradigm to assess child focused attention (Gaertner et al., 2008; Ruff & Lawson, 1990). Each child was provided with 13 colorful blocks and shown how to play with them. They were then allowed to play while the experimenter sat aside without interacting with the child. The session lasted up to 4 min, with 55% of children reaching the maximum duration (mean playtime = 3.4 min, range = 1.25–4 min). Children’s focused attention was assessed by observing their engagement with task-related toys, specifically noting how well they attended, concentrated, and oriented toward these items. Indicators of high attention included prolonged, steady gaze, intent facial expression, close visual proximity, and active manipulation of the materials. Conversely, lower levels of attention were reflected in a lack of visual orientation, frequent off-task glances, and passive or repetitive handling of the materials (Egotubov et al., 2023).

Using a coding system developed to assess toddlers’ focused attention (Gaertner et al., 2008), video recordings were segmented into 5-second intervals and micro-coded, with each interval receiving a focused attention score. Attention scores ranged from 1 (*None*- child did not pay attention to the task or only gave brief, unfocused glances at the task object attention) to 4 (*Very High*- child appeared deeply absorbed and focused on the task, showing intense interest and prolonged manipulation of the materials; Interrater reliability, calculated using ICC, was 0.86, *F*(34, 34) = 7.10, *p* <.000). Each child’s overall score represented the proportion of intervals where they exhibited high attention. To enhance construct validity, we assessed whether the observed focused attention scores correlated with maternal reports of children’s attentional focusing skills, as measured by the Early Childhood Behavior Questionnaire (ECBQ; Putnam et al., 2006), and found a significant positive association (*r*_(129)_ = 0.33, *p* <.01), suggesting that these measured tap into a similar construct to some extent.

### Covariates

Maternal years of education were controlled for, as previous studies have shown associations between education level and both depression (Ladin, 2008) and ADHD (Biederman et al., 2008). Additionally, child sex was controlled to account for potential sex differences in depressive symptoms and focused attention in children (Salk et al., 2017). Finally, child age was included as a covariate, as focused attention has been shown to improve with age (Ruff & Lawson, 1990).

### Missing Data

Overall, 99 families had data available across all time points, representing 63% of the sample, with an average missing data rate of 6/9% (see Tables 1 and 2 for the percentage of missing data for each variable). Little’s test was performed using the R package ‘naniar’ version 1.0.0 (Tierney et al., 2024), yielding non-significant results ($\:\chi\:$^2^_(45)_ *=* 59.20, *p* =.076), indicating that the missing data are missing completely at random. All missing data were completed using multiple imputations via the ‘mice’ R package (version 3.15.0; van-Buuren & Groothuis-Oudshoorn, 2011), with each imputed dataset estimated separately using the predictive mean-matching method.

### Statistical Analysis

Analyses were performed using R software version 6.0.421 (R Core Team, 2023). Preliminary analyses included assessing group differences, calculating bivariate correlations between study variables, and examining correlations with demographic variables. To test our hypothesis, we conducted path analyses using the ‘semTools’ R package (version 0.5-6; Jorgensen et al., 2022), estimating a saturated model in which child depressive symptoms and child focused attention were regressed on the study covariates, MDS measured at T1-T3, maternal ADHD symptoms at T1, and the interaction terms “T1_MDS × T1_Maternal ADHD” and “T2_MDS × T1_Maternal ADHD.” All independent variables were mean-centered. Significant interactions were further explored by estimating simple slopes at the mean and at ± 1 standard deviation of child depressive symptoms and child focused attention and Johnson-Neyman regions of significance analysis (Johnson & Fay, 1950) via the ‘interactions’ R package (version 1.1.5; Long, 2019).

## Results

### Preliminary Analysis and Descriptive Statistics

Descriptive statistics and intercorrelations between all study variables are presented in Table 2. As shown, there were significant positive correlations between MDS across all time points. Additionally, maternal ADHD symptoms were positively associated with MDS at T1-T3 and with child depressive symptoms. Child depressive symptoms were also positively correlated with MDS, but only at T2, with no significant correlations at other time points. Furthermore, at T3, child age was negatively associated with MDS and positively associated with child focused attention.

**Table 2:** Means, standard deviations, and correlations with confidence intervals

Variable	M	SD	% Missing	1	2	3	4	5	6	7	8
1. Maternal Education Years	15.25	2.96	1%								
2. Child Sex	1.51	0.50	13%	0.07							
				[-0.10, 0.23]							
3. Child Age T3	26.14	1.98	6%	− 0.02	0.01						
				[-0.18, 0.14]	[-0.17, 0.18]						
4. Maternal ADHD Symptoms	8.45	4.29	1%	− 0.01	− 0.02	− 0.01					
				[-0.17, 0.15]	[-0.19, 0.15]	[-0.18, 0.15]					
5. MDS T1	5.43	4.44	2%	− 0.05	0.05	0.02	0.51**				
				[-0.21, 0.11]	[-0.12, 0.22]	[-0.14, 0.18]	[0.38, 0.62]				
6. MDS T2	3.74	3.40	6%	− 0.01	− 0.07	− 0.16	0.16*	0.35**			
				[-0.17, 0.16]	[-0.24, 0.10]	[-0.32, 0.01]	[0.00, 0.32]	[0.20, 0.48]			
7. MDS T3	4.29	3.91	8%	0.01	0.03	− 0.18*	0.26**	0.35**	0.54**		
				[-0.16, 0.17]	[-0.15, 0.20]	[-0.34, − 0.02]	[0.09, 0.40]	[0.20, 0.49]	[0.41, 0.65]		
8. Child Depressive Symptoms	2.13	2.18	13%	− 0.10	0.18	0.02	0.26**	0.16	0.25**	0.12	
				[-0.26, 0.07]	[-0.00, 0.35]	[-0.15, 0.19]	[0.09, 0.41]	[-0.01, 0.32]	[0.08, 0.40]	[-0.05, 0.29]	
9. Child Focused Attention	0.55	0.24	11%	− 0.07	0.03	0.18*	0.03	0.01	− 0.13	− 0.08	− 0.14
				[-0.23, 0.10]	[-0.15, 0.20]	[0.01, 0.33]	[-0.14, 0.20]	[-0.16, 0.18]	[-0.30, 0.04]	[-0.25, 0.09]	[-0.31, 0.03]

*Note. M* and *SD* are used to represent mean and standard deviation, respectively. Values in square brackets indicate the 95% confidence interval for each correlation. * indicates *p* <.05. ** indicates *p* <.01

### Path Model

**Fig. 1:**
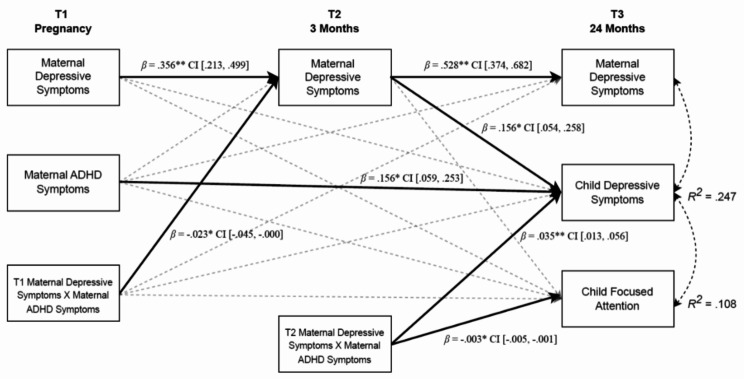
A path model predicting interactive effects between MDS, maternal ADHD, child depressive symptoms and child focused attention. **Note** Control variables are included in the analysis but are not displayed in the figure to enhance simplicity and clarity. * *p* <.05, **p <.001.

To test the study hypotheses, we estimated a path model in which child depressive symptoms and child focused attention at T3 were regressed on the study covariates, MDS measured at T1-T3, maternal ADHD symptoms at T1, and the interaction terms “T1_MDS × T1_Maternal ADHD” and “T2_MDS × T1_Maternal ADHD” (Fig. 1, see supplementary materials for full model results).

Higher maternal ADHD symptoms (*β =* 0.156, *SE* = 0.05, *p* =.002) and higher MDS at T2 (*β =* 0.156, *SE* = 0.05, *p* =.003) predicted higher child depressive symptoms at T3. However, neither maternal ADHD symptoms nor MDS significantly predicted child focused attention. Nevertheless, the interaction between maternal ADHD symptoms and MDS at T2 predicted child depressive symptoms (*β =* 0.035, *SE* = 0.01, *p* =.002) and child focused attention (*β =-* 0.003, *SE* = 0.00, *p* =.014). In addition, the interaction between maternal ADHD symptoms and MDS at T1 predicted MDS at T2 (*β = −.0*23, *SE* = 0.01, *p* =.048); see supplementary materials for post-hoc analysis). It should be noted that MDS demonstrated stability across measurements.

#### Simple Slopes and Regions of Significance Analyses

To further explore the significant interaction effects on both child depressive symptoms and focused attention, simple slopes were calculated for low (-1 SD), average, and high (+ 1 SD) levels of maternal ADHD symptoms.

**Fig. 2:**
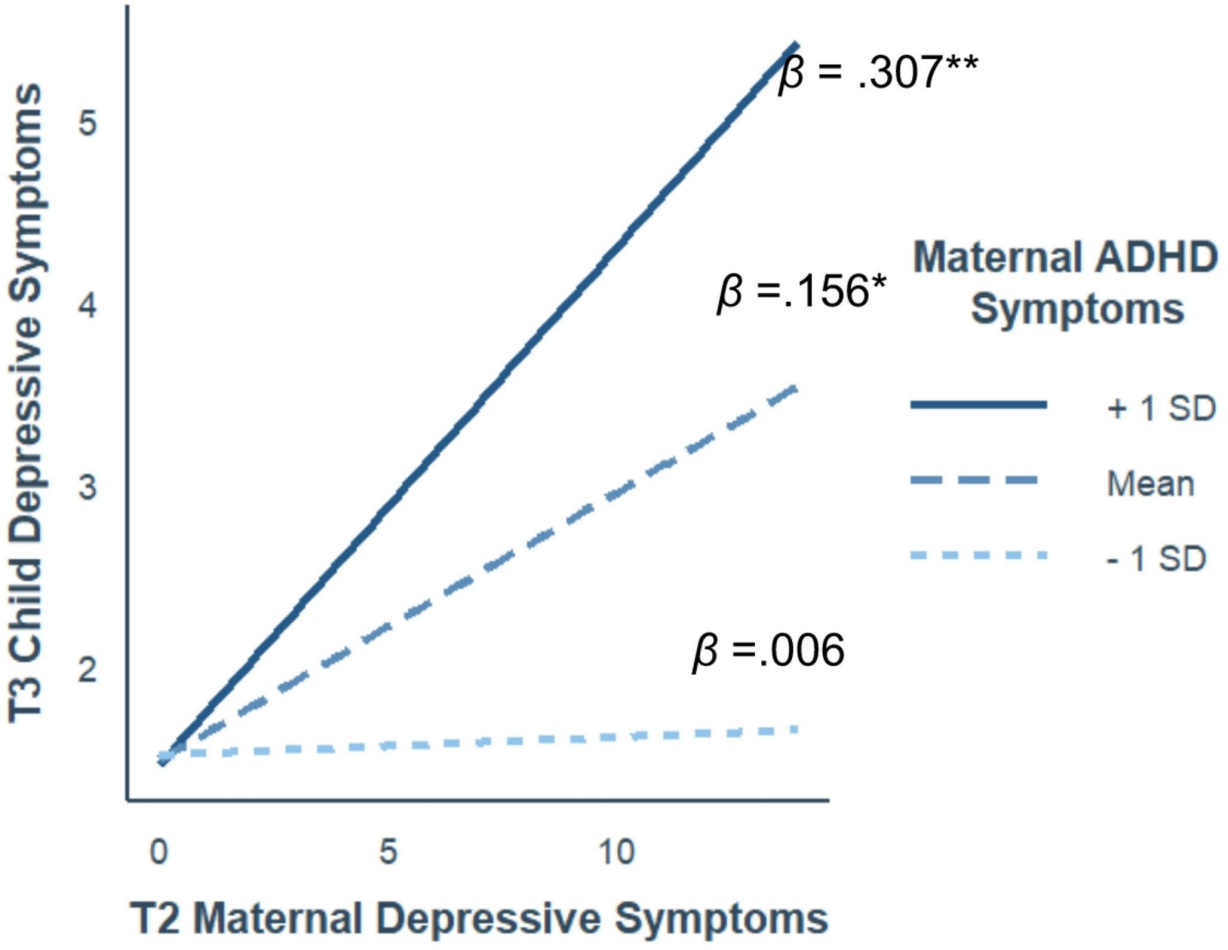
The links between MDS at 3 months and child depressive symptoms as a function of maternal ADHD symptoms. **Note** Axes represent nonstandardized scores; *β*s represent the standardized simple slopes. * *p* <.05, **p <.001

**Fig. 3:**
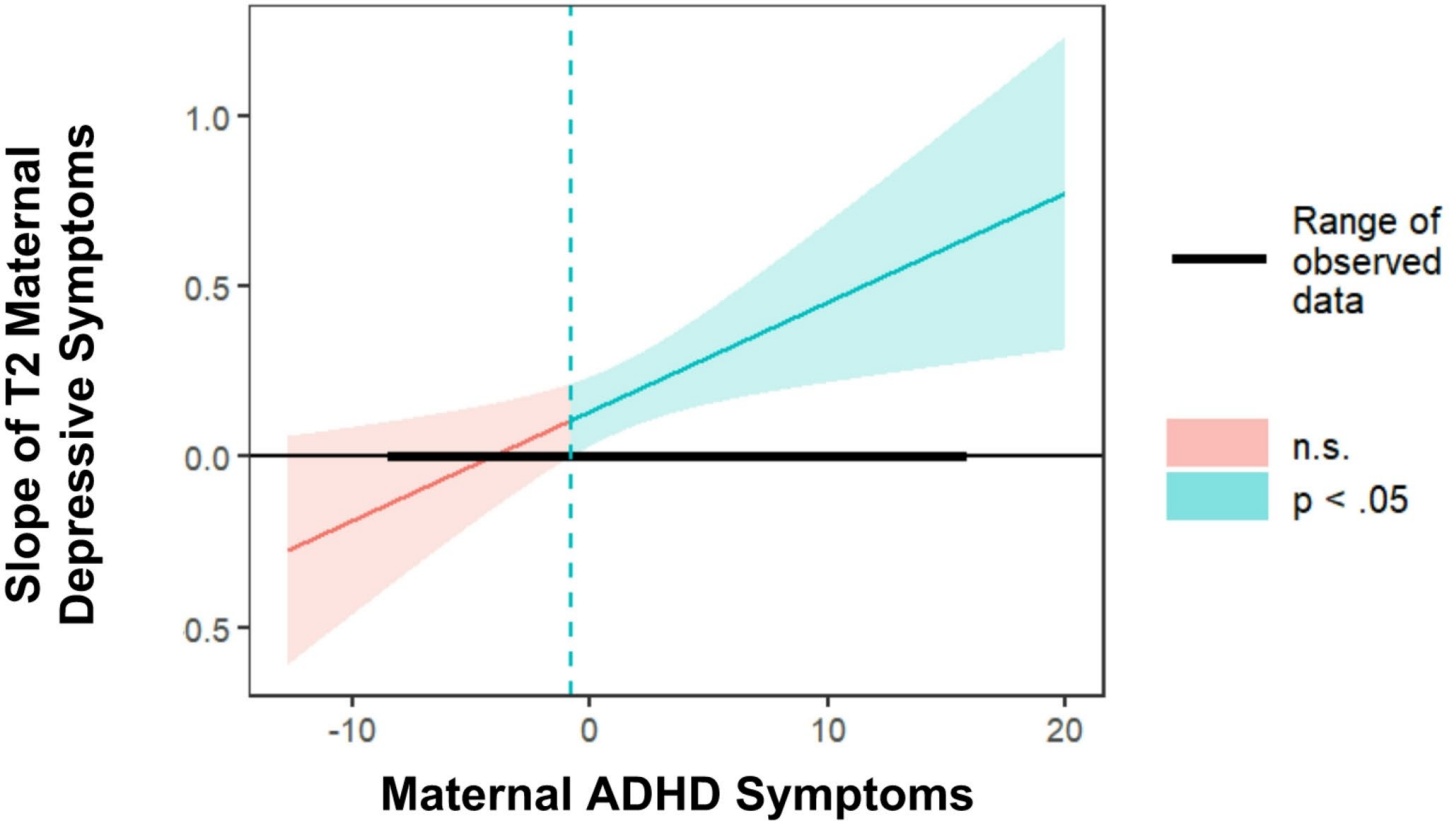
Johnson-Neyman analysis results, depicting the point-estimate of the slope linking MDS and child depressive symptoms as a function of maternal ADHD symptoms. **Note** Analysis was conducted with a 95% confidence interval. values above – 0.79 (dotted line) are significantly different from zero

For *child depressive symptoms* (Fig. 2), the association between T2_MDS and child depressive symptoms, was positive and significant at average and high levels of maternal ADHD symptoms (*β =* 0.156, 95% CI [0.05, 0.26], *p* =.003 and *β =* 0.307, 95% CI [0.16, 0.45], *p* <.000, respectively), but not at low levels of maternal ADHD symptoms (*β =* 0.006, 95% CI [-0.13, 0.14], *p* =.928). Johnson-Neyman analysis regions of significance analysis revealed that the effect of MDS on child depressive symptoms is significant when maternal ADHD symptoms are − 0.79 or higher (54% of the sample; Fig. 3).

**Fig. 4:**
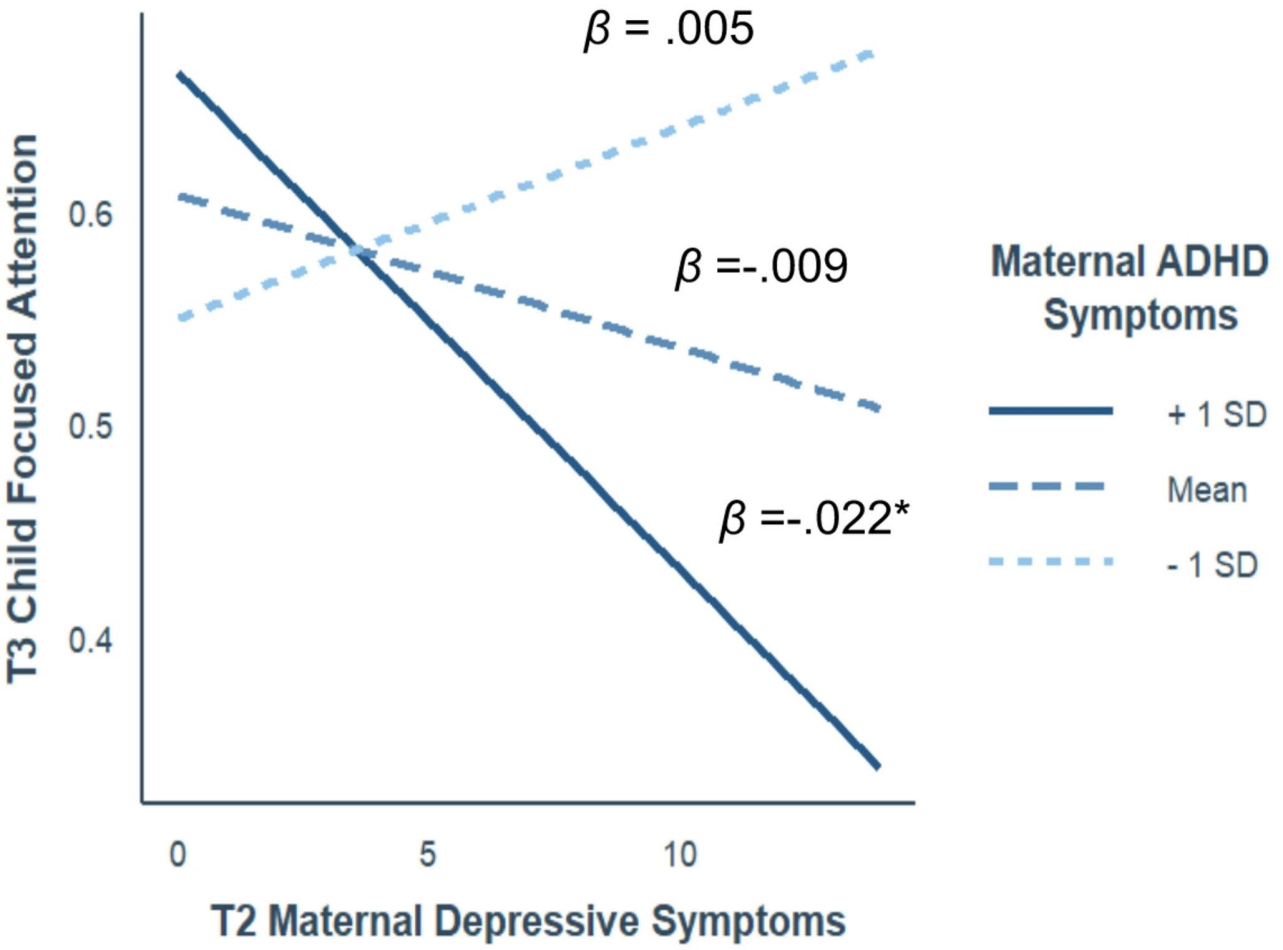
The links between MDS at 3 months and child focused attention as a function of maternal ADHD symptoms. **Note** Axes represent nonstandardized scores; *β*s represent the standardized simple slopes. * *p* <.05, **p <.001

**Fig. 5:**
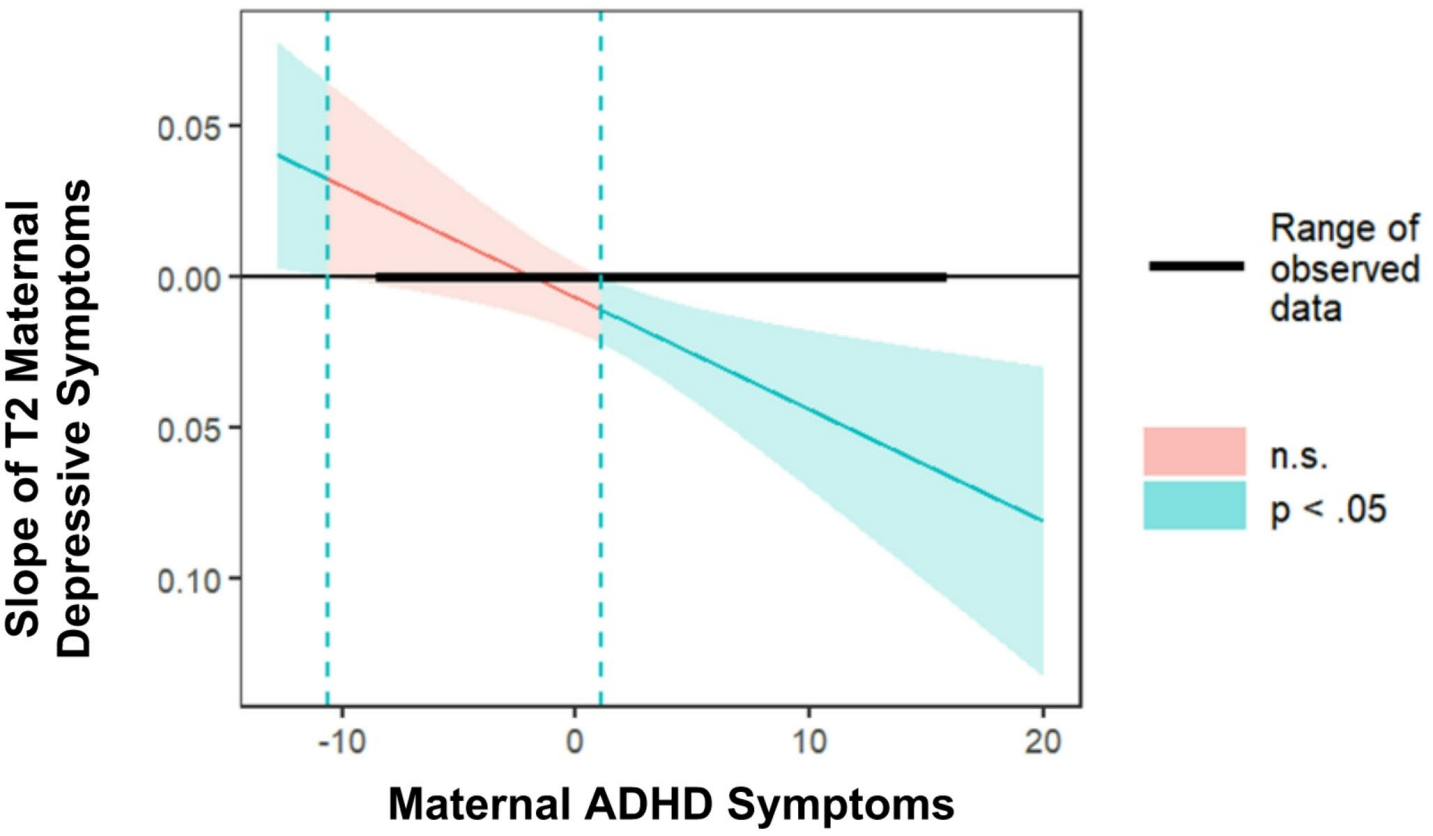
Johnson-Neyman analysis results, depicting the point-estimate of the slope linking MDS and child focused attention as a function of maternal ADHD symptoms. **Note** Analysis was conducted with a 95% confidence interval. values above 1.07 (dotted line) are significantly different from zero

For *child focused attention* (Fig. 4), the link between T2_MDS and child focused attention was negative and significant only when maternal ADHD symptoms were high (*β = −.0*22, 95% CI [-0.04, -0.01], *p* =.005), and non-significant at average or low levels of maternal ADHD symptoms (*β = −.0*09, 95% CI [-0.02, 0.01], *p* =.115 and *β =* 0.005, 95% CI [-0.01, 0.02], *p* =.550, respectively). Johnson-Neyman interval revealed that the effect of MDS on child focused attention was significant when maternal ADHD symptoms were 1.07 or higher (35% of the sample; Fig. 5).

## Discussion

Maternal mental health plays a critical role in shaping child development, with depression (Goodman et al., 2011) and ADHD symptoms (Brammer et al., 2018) emerging as particularly influential factors. Despite this, the unique and combined impacts of maternal depressive symptoms and ADHD on children’s outcomes, especially in early childhood, remain underexplored (Brammer et al., 2018; Smit et al., 2021). Therefore, this study sought to investigate the interplay between maternal depression and ADHD symptoms, two interrelated yet understudied risk factors, on children’s depressive symptoms and focused attention abilities at age 24 months. Results showed that maternal ADHD and depression symptoms had direct and interactive effects on children’s depressive symptoms, while children’s focused attention was predicted solely by the interaction effect. These findings highlight the combined impact of maternal depression and ADHD, suggesting that their co-occurrence is linked to greater child depressive symptoms and attention difficulties than either condition alone.

With respect to child depressive symptoms, our findings reveal that they were predicted by maternal ADHD symptoms during pregnancy, maternal depression at 3 months postpartum, and the interaction between these two factors. The positive association between maternal and child depressive symptoms aligns with previous research and the integrative model for the transmission of risk to children of depressed mothers (Goodman & Gotlib, 1999; Hammen et al., 2012; Murray et al., 2011). Maternal ADHD symptoms were also positively associated with child depressive symptoms. While limited studies have directly examined this link, one study found that parental ADHD was related to child psychopathology, encompassing internalizing, externalizing, and ADHD symptoms, though this was assessed only cross-sectionally (Humphreys et al., 2012). Furthermore, prior research has demonstrated that parental ADHD impacts parenting behaviors (Friedrich et al., 2017; Johnston & Chronis-Tuscano, 2017), and difficulties in parent-child interactions, such as negative parenting behaviors and stressful environments, have been associated with child depression (Humphreys et al., 2013). This suggests that maternal ADHD may contribute to child depressive symptoms through negative and maladaptive parenting practices.

Finally, the interaction between maternal ADHD and depressive symptoms was found to predict child depressive symptoms, with MDS at 3 months being significantly linked to child depressive symptoms only when maternal ADHD symptoms were at moderate to high levels. This finding can be contextualized using the heuristic model proposed by Johnston and Chronis-Tuscano (2017), which outlines how parental ADHD symptoms may interact with other parental psychopathologies to influence parenting practices and child mental health outcomes. Parents with both depression and ADHD often encounter overlapping challenges, such as difficulties in executive functioning (Bain, 2018), reduced quality of life, greater interpersonal difficulties (Huang et al., 2022), more severe depressive symptoms, and higher hospitalization rated (Biederman et al., 2008). Regarding the impact on children, while parental ADHD and depression independently predict child psychopathology (Humphreys et al., 2012), co-occurrence leads to heightened risks, such as increased emotional and behavioral impairments (Powell et al., 2021). Building on these findings and the heuristic model, we can conclude that the combined impact of maternal ADHD and depressive symptoms may create a uniquely challenging environment, amplifying child vulnerability and risks for child depressive symptoms (Goodman & Gotlib, 1999).

Regarding child focused attention, we found that only the interaction between maternal depression at 3 months and maternal ADHD predicted child focused attention, rather than each factor individually. Previous findings found parental ADHD to be related to child attentional difficulties (Humphreys et al., 2012; Johnston & Chronis-Tuscano, 2017), and maternal depressive symptoms to be associated with poor sustained attention in children (Wang & Dix, 2017), which highlights how maternal distress impacts children’s focused attention. Our study extends these findings by suggesting that the combined influence of maternal depression and ADHD symptoms has a more significant effect on child focused attention than either factor alone. This aligns with literature on co-occurring parental psychopathologies, which suggests that multiple parental disorders have an additive impact on child psychological outcomes (McLaughlin et al., 2012). Furthermore, research has shown that children of mothers with comorbid depression and antisocial personality disorder experience more socioemotional difficulties, alongside negative parenting behaviors (Russotti et al., 2023). Overall, our results support the notion that the interactive effect of maternal depression and ADHD has a more profound and complex influence on child outcomes than the individual effects of these symptoms. One possible mechanism underlying this finding is through genetic influences. ADHD and depression, which frequently co-occur (Choi et al., 2022), also share a common foundation in the serotonergic and dopaminergic systems (Faraone & Larsson, 2019; Johnston & Chronis-Tuscano, 2015; Levinson, 2006). Furthermore, both conditions are heritable (Larsson et al., 2014; Sawyer et al., 2019), which can explain how maternal ADHD and depressive symptoms might jointly influence child attention. Another possible mechanism involves how the combined maternal ADHD and depressive symptoms may lead to inconsistent parenting behaviors, reduced sensitivity, lower involvement, increased intrusiveness, and heightened negative emotions (Field, 2010; Friedrich et al., 2017; Smit et al., 2021). Such maladaptive behaviors could disrupt the regulatory processes essential for focused attention. Mothers experiencing ADHD and depressive symptoms often struggle with executive functioning challenges (Bain, 2018), and face more severe depressive symptoms (Biederman et al., 2008). These difficulties may hinder their ability to provide consistent scaffolding or to help their child regulate emotions and behavior during interactions. Moreover, the interplay of these conditions can lead to increased frustration and difficulty managing a child’s attentional challenges. In such cases, mothers might become disengaged or exhibit hostility, further undermining the child’s self-regulation and impairing a child’s ability to concentrate and maintain attention over time.

It is important to note that contrary to our hypothesis, MDS during pregnancy did not predict child depressive symptoms or focused attention, while postpartum MDS did. Prior research highlights that both prenatal and postpartum maternal depression can significantly impact child development (Pearson et al., 2013). While prenatal depression influences outcomes through biological mechanisms (Waters et al., 2014), studies have demonstrated unique contributions of postpartum depression as well (Barker et al., 2011; Ross et al., 2020). For example, it was found that while both prenatal and postnatal depression were associated with increased externalizing problems and a slight decrease in verbal IQ, only postpartum depression was linked to increased internalizing problems (Barker et al., 2011). Similarly, another study demonstrated that postnatal (buy not prenatal) depressive symptoms were associated with poorer parent-reported child attention (Ross et al., 2020). These findings align with the interpersonal stress transmission model (Hammen et al., 2004), which depicts how maternal depression may influence child outcomes through stressful interpersonal processes such as parenting. Therefore, the associations between MDS and child outcome, can be understood through the role of maternal depression in parenting behaviors and parent-child interactions (Hakanen et al., 2019; Lovejoy et al., 2000), which in turn affect early childhood development (Wang & Dix, 2017). Consequently, our findings suggest that postpartum MDS may exert a more pronounced impact on children’s outcomes due to its influence on parenting practices. It is important to acknowledge that parenting was not measured in this study, and the proposed hypothesis remains speculative. Testing this hypothesis would require a direct assessment of parenting characteristics.

### Limitations and Future Directions

Our findings must be interpreted with caution due to several limitations. First, all maternal measures and child depressive symptoms were based on self-reports by mothers, which may introduce response bias due to subjective perceptions. Additionally, examining both caregivers could enhance our understanding of how parental psychopathology influences parenting and child outcomes. Prior research suggests that fathers may play a protective role in buffering the effects of maternal psychopathology on children (Martin et al., 2022). Moreover, couples with shared psychopathology may communicate more positively when managing disruptive behaviors (Wymbs et al., 2017). Utilizing objective assessments, observational data, or input from other caregivers (e.g., fathers) could strengthen the study design and improve result interpretation. Second, the sample was drawn primarily from relatively educated, low-risk families, limiting the generalizability of our findings to more diverse or high-risk populations. In these populations, additional stressors may play a role and interact with the studied variables, potentially influencing the observed patterns (Russell et al., 2016; Sullivan et al., 2019). Third, the relatively small sample size limits the study’s ability to examine complex models that require greater statistical power. Lastly, maternal ADHD symptoms were assessed only during pregnancy, limiting the understanding of their evolution over time using more contemporary analytical approaches that model changes within and between subjects through time (Berry & Willoughby, 2017). Although ADHD symptoms persist throughout the lifespan (Turgay et al., 2012), and self-reports of ADHD diagnoses provided consistency, adding follow-up measures postpartum could have illuminated other potential pathways through which maternal ADHD symptoms might influence child depressive symptoms and focused attention.

Future research should incorporate longitudinal assessments of both maternal ADHD symptoms and child outcomes to explore dynamic changes and their long-term implications. Additionally, studies indicate that maternal parenting behaviors mediate the relationship between maternal psychopathology and child outcomes (Klemp et al., 2023). Therefore, future investigations should also examine how maternal depression and ADHD interact to shape parenting practices, which may in turn influence child depressive symptoms and focused attention. This approach would provide a deeper understanding of the mechanisms through which maternal psychopathology impacts child development. Finally, other familial or child characteristics, such as temperament, physiological and neurological functioning, or sleep problems, may play a role in the relationship between maternal depression, ADHD, and child outcomes (Dai et al., 2022; Goodman & Gotlib, 1999; Lionetti et al., 2022; Yang et al., 2020). Future research should consider these factors to provide a more comprehensive understanding of how maternal psychopathology influences child development.

### Conclusion and Clinical Implications

The current study aimed to explore the relationship between maternal depression and ADHD symptoms and how they are associated with children’s depressive symptoms and focused attention during toddlerhood. Our findings expand the literature by demonstrating longitudinally that the combination of maternal depression and ADHD predicts impairments in children’s emotional and cognitive development. These results underscore the importance of clinical diagnoses and highlight the need to prioritize support for children of parents with comorbid psychopathology. Screening families for maternal ADHD and depression is crucial to ensuring that both parents and children receive the necessary interventions and support.

## Electronic Supplementary Material

Below is the link to the electronic supplementary material.

Supplementary Material 1

## Data Availability

Summary aggregate level data and analysis code for this study can be made available upon reasonable request to the corresponding author.
