# Supplementary material for: The Interplay between Maternal Depression and ADHD Symptoms in Predicting Emotional and Attentional Functioning in Toddlerhood
Source: Res Child Adolesc Psychopathol. 2025 May 19;53(8):1231–47. doi: 10.1007/s10802-025-01332-y (PMC12357811; doi:10.1007/s10802-025-01332-y)
Supplement: Supplementary file 1 — Supplementary Material 1 [file 10802_2025_1332_MOESM1_ESM.docx]

#

# **Results**

**Full Path Model Results**

**Table S1**

*Regression Results from Path Model in Predicting Child Depressive Symptoms and Child Focused Attention*

| **Outcome** | **Predictor** | **β** | **SE** | **95% CI (Lower, Upper)** | **p-value** |
| --- | --- | --- | --- | --- | --- |
| T3 Child depressive symptoms |  |  |  |  |  |
|  | T1 Maternal depressive symptoms | -.055 | 0.053 | (-0.160, 0.049) | .299 |
|  | T2 Maternal depressive symptoms | .156 | 0.052 | (0.054, 0.258) | .007 ** |
|  | T1 Maternal ADHD symptoms | .156 | 0.055 | (0.059, 0.253) | .008 ** |
|  | T1 Maternal depressive symptoms **×** T1 Maternal ADHD symptoms | .007 | 0.007 | (-0.006, 0.020) | .287 |
|  | T2 Maternal depressive symptoms **×** T1 Maternal ADHD symptoms | .035 | 0.012 | (0.013, 0.056) | .005 ** |
|  | Maternal education | -.077 | 0.062 | (-0.198, 0.044) | .210 |
|  | Child sex | .550 | 0.268 | (0.025, 1.075) | .040 * |
|  | T3 Child age | .019 | 0.095 | (-0.167, 0.205) | .842 |
| T3 Child focused attention |  |  |  |  |  |
|  | T1 Maternal depressive symptoms | .002 | 0.008 | (-0.013, 0.017) | .789 |
|  | T2 Maternal depressive symptoms | -.006 | 0.006 | (-0.018, 0.007) | .368 |
|  | T1 Maternal ADHD symptoms | -.002 | 0.006 | (-0.014, 0.010) | .777 |
|  | T1 Maternal depressive symptoms **×** T1 Maternal ADHD symptoms | .000 | 0.001 | (-0.002, 0.003) | .790 |
|  | T2 Maternal depressive symptoms **×** T1 Maternal ADHD symptoms | -.003 | 0.001 | (-0.005, -0.001) | .009 ** |
|  | Maternal education | -.007 | 0.008 | (-0.023, 0.009) | .374 |
|  | Child sex | .016 | 0.029 | (-0.039, 0.072) | .563 |
|  | T3 Child age | .021 | 0.010 | (0.002, 0.040) | .032 * |
| T3 Maternal depressive symptoms |  |  |  |  |  |
|  | T1 Maternal depressive symptoms | .068 | 0.073 | (-0.075, 0.211) | .353 |
|  | T2 Maternal depressive symptoms | .528 | 0.070 | (0.374, 0.682) | <.001 *** |
|  | T1 Maternal ADHD symptoms | .129 | 0.073 | (-0.015, 0.273) | .079† |
|  | T1 Maternal depressive symptoms **×** T1 Maternal ADHD symptoms | .015 | 0.010 | (-0.005, 0.035) | .149 |
|  | T2 Maternal depressive symptoms **×** T1 Maternal ADHD symptoms | .028 | 0.014 | (-0.000, 0.056) | .051† |
|  | Maternal education | .013 | 0.110 | (-0.203, 0.229) | .906 |
|  | Child sex | .189 | 0.377 | (-0.549, 0.928) | .615 |
|  | T3 Child age | -.196 | 0.091 | (-0.374, -0.018) | .031 * |
| T2 Maternal depressive symptoms |  |  |  |  |  |
|  | T1 Maternal depressive symptoms | .356 | 0.073 | (0.213, 0.499) | <.001 *** |
|  | T1 Maternal ADHD symptoms | -.019 | 0.064 | (-0.144, 0.105) | .763 |
|  | T1 Maternal depressive symptoms **×** T1 Maternal ADHD symptoms | -.023 | 0.011 | (-0.045, -0.000) | .044 * |
|  | Maternal education | .049 | 0.087 | (-0.123, 0.220) | .578 |
|  | Child sex | -.228 | 0.372 | (-0.958, 0.501) | .540 |
|  | T3 Child age | -.220 | 0.137 | (-0.488, 0.048) | .108 |

**Note**: SE = Standard Error; CI = Confidence Interval. †*p* < .10. **p* < .05. ***p* < .01. ****p* < .001.

**Simple Slopes and Regions of Significance Analyses**

***Maternal Depression Symptoms***

We also analyzed the simple slopes for the interaction between maternal ADHD symptoms and depressive symptoms at T1 in predicting depressive symptoms at T2. The association between MDS at T1 and T2 was positive and significant across low, average, and high levels of maternal ADHD symptoms (*β =* .454, 95% CI [0.24, 0.67], *p* < .000, *β =* .356, 95% CI [0.21, 0.50], *p* < .000, and *β =* .258, 95% CI [0.13, 0.38], *p* < .000, respectively), suggesting that at all levels of ADHD symptoms MDS at T1 predicted higher MDS at T3.
